# Acidification, not carbonation, is the major regulator of carbon fluxes in the coccolithophore *Emiliania huxleyi*

**DOI:** 10.1111/nph.13885

**Published:** 2016-02-25

**Authors:** Dorothee M. Kottmeier, Sebastian D. Rokitta, Björn Rost

**Affiliations:** ^1^ Alfred Wegener InstituteHelmholtz Centre for Polar and Marine ResearchAm Handelshafen 1227570BremerhavenGermany

**Keywords:** calcification, CO_2_‐concentrating mechanism, life‐cycle stages, membrane‐inlet mass spectrometry, ocean acidification, pH, photosynthesis

## Abstract

- A combined increase in seawater [CO_2_] and [H^+^] was recently shown to induce a shift from photosynthetic HCO_3_^−^ to CO_2_ uptake in *Emiliania huxleyi*. This shift occurred within minutes, whereas acclimation to ocean acidification (OA) did not affect the carbon source.
- To identify the driver of this shift, we exposed low‐ and high‐light acclimated *E. huxleyi* to a matrix of two levels of dissolved inorganic carbon (1400, 2800 μmol kg^−1^) and pH (8.15, 7.85) and directly measured cellular O_2_, CO_2_ and HCO_3_^−^ fluxes under these conditions.
- Exposure to increased [CO_2_] had little effect on the photosynthetic fluxes, whereas increased [H^+^] led to a significant decline in HCO_3_^−^ uptake. Low‐light acclimated cells overcompensated for the inhibition of HCO_3_^−^ uptake by increasing CO_2_ uptake. High‐light acclimated cells, relying on higher proportions of HCO_3_^−^ uptake, could not increase CO_2_ uptake and photosynthetic O_2_ evolution consequently became carbon‐limited.
- These regulations indicate that OA responses in photosynthesis are caused by [H^+^] rather than by [CO_2_]. The impaired HCO_3_^−^ uptake also provides a mechanistic explanation for lowered calcification under OA. Moreover, it explains the OA‐dependent decrease in photosynthesis observed in high‐light grown phytoplankton.

## Introduction

Coccolithophores are unicellular calcareous algae that take a dual role in global carbon cycling. During photosynthesis, carbon dioxide (CO_2_) is fixed into organic matter, leading to a net decrease in dissolved inorganic carbon (DIC) and CO_2_ from seawater. In the process of calcification, calcium carbonate (CaCO_3_) is precipitated, which results in lowered DIC and alkalinity, thus elevated CO_2_ levels. *Emiliania huxleyi* is the most abundant coccolithophore in the present‐day ocean with a distribution from tropical to subpolar waters (Winter *et al*., 2013). The species is able to form extensive blooms (Brown & Yoder, 1994; Sadeghi *et al*., 2012), which are often associated with a shallow mixed‐layer depth and high irradiances (Nanninga & Tyrrell, 1996; Raitsos *et al*., 2006). As one of the most important pelagic calcifiers, *E. huxleyi* has been a major focus of oceanographic research over the last decades, in particular with respect to ocean acidification (OA; e.g. Rost & Riebesell, 2004; Raven & Crawfurd, 2012).

As the ocean takes up anthropogenic CO_2_, levels of HCO_3_^−^ and CO_2_ increase, whereas pH and levels of CO_3_^2−^ decrease (Wolf‐Gladrow *et al*., 1999). These changes in carbonate chemistry are often summarized as OA, but strictly speaking this phenomenon comprises carbonation (i.e. increased [CO_2_] and [HCO_3_^−^]) as well as acidification (i.e. increased [H^+^]/lowered pH). With a few exceptions, investigations of OA effects on *E. huxleyi* and other coccolithophores showed stimulated or unaffected production rates of particulate organic carbon (POC, i.e. biomass), with concomitantly impaired or unaffected production rates of particulate inorganic carbon (PIC, i.e. CaCO_3_; see Raven & Crawfurd, 2012, for overview). Some of the observed diversity in the OA responses could be attributed to genetic variability; but more importantly environmental factors such as irradiance were shown to modulate OA effects (Nielsen, 1997; Zondervan *et al*., 2002; van de Poll *et al*., 2007; Feng *et al*., 2008; Rokitta & Rost, 2012; Sett *et al*., 2014; Xu & Gao, 2015). OA responses are typically measured after acclimation to altered conditions over several generations, allowing cells to adjust their metabolism. A study by Barcelos e Ramos *et al*. (2010) demonstrated that the OA‐induced changes in cellular POC and PIC production are already evident after a few hours, indicating that OA effects are relatively immediate. In order to identify the drivers causing the OA responses in *E. huxleyi*, Bach and co‐workers disentangled the effects of carbonation and acidification by acclimating cells to artificial carbonate chemistry conditions (Bach *et al*., 2011, 2013). In these experiments, POC and PIC production were shown to be stimulated by carbonation, but inhibited by acidification.

In order to improve our understanding of *E. huxleyi*'s response to OA, it is important to assess which cellular processes are affected by carbonation, acidification or the combination of both. *Emiliania huxleyi* is known to use CO_2_ and HCO_3_^−^ as external inorganic carbon (C_i_) sources of photosynthesis, but the estimated proportions of CO_2_ uptake differ between studies and depend on the applied methods and assay conditions (e.g. Sikes *et al*., 1980; Herfort *et al*., 2002; Trimborn *et al*., 2007; Kottmeier *et al*., 2014). The increase in POC production after acclimation to OA is often attributed to the higher aqueous CO_2_ levels, which are thought to directly increase the diffusive CO_2_ supply at the CO_2_‐fixing enzyme Ribulose‐1,5‐bisphosphate‐carboxylase/‐oxygenase (RubisCO; Raven & Johnston, 1991; Rokitta & Rost, 2012; Stojkovic *et al*., 2013). A recent study demonstrated that the fraction of photosynthetic CO_2_ uptake relative to active HCO_3_^−^ uptake is indeed strongly increased under high [CO_2_]/low pH (Kottmeier *et al*., 2014). This switch in the C_i_ source occurred at short timescales of seconds to minutes, whereas the acclimation to OA did not significantly affect the C_i_ source. Thus, the beneficial OA effect seems to be directly caused by the changing carbonate chemistry rather than by changes in the expression of genes related to the CO_2_‐concentrating mechanism (CCM). The inhibitory effect of OA on PIC production is often attributed to changes in electrochemical gradients under high [H^+^] and the associated costs of H^+^ removal (Anning *et al*., 1996; Berry *et al*., 2002; Suffrian *et al*., 2011; Taylor *et al*., 2011). Tracer studies found HCO_3_^−^ to be the major external C_i_ source for calcification (Paasche, 1964; Sikes *et al*., 1980; Buitenhuis *et al*., 1999; Herfort *et al*., 2002; Rost *et al*., 2002), and it was suggested that increased H^+^ levels also affect HCO_3_^−^ uptake mechanisms (Fukuda *et al*., 2014). Despite the gained knowledge on cellular processes, relatively little is known about the differential effects of carbonation and acidification on photosynthesis, calcification and their underlying C_i_ supply.

**Figure 1:**
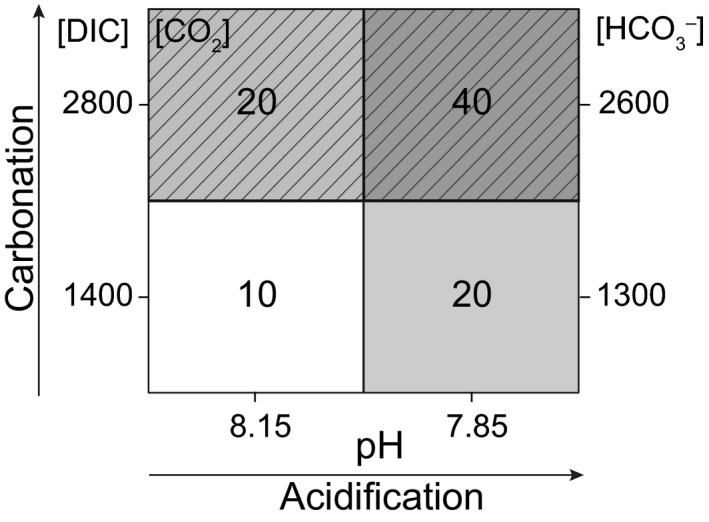
Decoupled carbonate chemistry during mass spectrometric measurements of cellular O_2_ and external inorganic carbon (C_i_) fluxes in *Emiliania huxleyi*. Applied conditions were: low dissolved inorganic carbon (DIC)/high pH (L_DIC_H_pH_; white); high DIC/high pH (H_DIC_H_pH_; dashed, light grey); low DIC/low pH (L_DIC_L_pH_; light grey); high DIC/low pH (H_DIC_L_pH_; dashed, dark grey). All concentrations are given in μmol kg^−1^.

In order to investigate the drivers causing the immediate shifts in the photosynthetic C_i_ source under high [CO_2_]/low pH (Kottmeier *et al*., 2014), here we measured the photosynthetic oxygen (O_2_) and C_i_ fluxes in direct response to carbonation, acidification and the combination of both. To this end, we acclimated both life‐cycle stages of *E. huxleyi* to present‐day carbonate chemistry and exposed them to a matrix of two DIC levels (1400 and 2800 μmol kg^−1^) and two pH values (8.15 and 7.85), yielding three different CO_2_ concentrations (~10, 20 and 40 μmol kg^−1^; Fig. 1). To further address the effect of energization, cells were acclimated to low and high photon flux densities (PFD; 50 and 400 μmol photons m^−2^ s^−1^), and fluxes were measured at two different PFD (180 and 700 μmol photons m^−2^ s^−1^).

## Materials and Methods

### Culture conditions

The calcifying diplont (diploid life‐cycle stage) *Emiliania huxleyi* (Lohmann) Hay and Mohler, strain RCC 1216, and its noncalcifying haplont, RCC 1217, were acclimated to low and high light levels (LL, 50 ± 30 μmol photons s^−1^ m^−2^; HL, 400 ± 30 μmol photons s^−1^ m^−2^) under present‐day carbonate chemistry and a 16 h : 8 h, light : dark‐cycle. Light was provided by daylight lamps (FQ 54W/965HO; OSRAM, Munich, Germany) and adjusted by measuring photon flux densities (PFD) inside water‐containing culturing bottles with a Walz Universal Light meter (ULM 500; Walz, Effeltrich, Germany) using a 4π‐sensor (US‐SQS/L).

Cells were grown as dilute‐batch cultures in sterile‐filtered North Sea seawater (0.2 μm, Sartobran 300; Sartorius AG, Gottingen, Germany) enriched with phosphate and nitrate (~7 and ~100* *μmol kg^−1^, respectively) as well as vitamins and trace metals according to F/2 (Guillard and Ryther, 1962). Culturing was performed in sterilized, gas‐tight 2‐l borosilicate bottles (Duran Group, Mainz, Germany), which were placed on roller tables to enable an homogenous cell suspension. Growth temperature was 15 ±1°C and was monitored by an Almemo 28–90 data logger (Ahlborn, Holzkirchen, Germany).

In all treatments, acclimations were performed under a CO_2_ partial pressure (*p*CO_2_) of 380 μatm (38.5 Pa), representing near‐present‐day conditions. The *p*CO_2_ was adjusted by pre‐aerating culture media with humidified, 0.2‐μm‐filtered air (Midisart 2000, PTFE; Sartorius AG), containing the desired *p*CO_2_. The gas mixture was created by a gas flow controller (CGM 2000; MCZ Umwelttechnik, Bad Nauheim, Germany) using pure CO_2_ (Air Liquide, Dusseldorf, Germany) and CO_2_‐free air (Air purification system; Parker, Kaarst, Germany). During the acclimation, head space inside the culture bottles was minimized to avoid outgassing effects. Carbonate chemistry was monitored based on total alkalinity (TA) measurements by potentiometric titration (Dickson, 1962; TitroLine alpha plus, measurement reproducibility ± 7 μmol kg^−1^; Schott Instruments, Mainz, Germany) and colorimetric DIC measurements with a QuAAtro autoanalyzer (measurement reproducibility ± 5 μmol kg^−1^; Seal Analytical, Norderstedt, Germany) in sterile‐filtered samples with the method of Stoll *et al*. (2001). Calculations of the carbonate system (CO2sys; Pierrot *et al*., 2006) were based on TA and DIC (Supporting Information Table S1). To monitor potential drifts of the carbonate chemistry on a daily basis, potentiometric measurements of pH_NBS_ were performed with a Metrohm pH meter (826 pH mobile; Metrohm, Filderstadt, Germany) with an electrode containing an integrated temperature sensor (Aquatrode Plus with Pt 1000, measurement reproducibility ± 0.01 pH units).

Cell growth was monitored by daily cell counting with a Coulter Counter (Beckman‐Coulter, Fullerton, CA, USA) and specific growth constants μ (d^−1^) were determined as μ = (log_e_ *c*_1_ – log_e_ *c*_0_) Δt^−1^ (*c*_1_ and *c*_0_, cell concentrations (cells ml^−1^); Δt, time interval (d)). In both life‐cycle stages, μ was more or less equal and significantly reduced in the low‐light acclimations (~0.7 d^−1^), confirming a light limitation in this treatment (Table S1). In the high‐light treatment, μ (~1.1 d^−1^) was at the upper range of previously reported growth constants for the same strain (Langer *et al*., 2009; Rokitta & Rost, 2012).

### Mass spectrometric flux measurements

Photosynthetic and respiratory O_2_ and C_i_ fluxes were measured with a mass spectrometer (Isoprime, GV Instruments, Manchester, UK) that was coupled to a cuvette via a gas‐permeable PTFE membrane (0.01 mm). This membrane‐inlet mass spectrometry (MIMS) technique uses the chemical disequilibrium between CO_2_ and HCO_3_^−^ during steady‐state photosynthesis to distinguish CO_2_ and HCO_3_^−^ uptake across the plasmalemma. Estimates of these fluxes were made following the equations of Badger *et al*. (1994). To include the process of calcification, we followed modifications introduced by Schulz *et al*. (2007).

The MIMS signals were calibrated for [CO_2_] by known additions of NaHCO_3_ into phosphoric acid (0.2 N), ensuring that all added DIC was quantitatively converted to CO_2_. Baseline values were obtained by adding sodium hydroxide (0.25 mmol l^−1^) into DIC‐free media, ensuring that any residual DIC was converted CO_3_^2−^. Calibration for [O_2_] was obtained by equilibrating medium with air (21% O_2_), followed by the addition of sufficient amounts of sodium dithionite (Merck) to quantitatively scavenge O_2_ (0% O_2_). MIMS signals were translated into [O_2_] by applying the O_2_ solubility constants of seawater (Weiss, 1970). All O_2_ signals were furthermore corrected for the machine‐inherent consumption.

Experiments were performed with cells in their exponential growth phase with maximal cell concentrations of 5 × 10^4^ cells ml^−1^ within 6–10 h after the start of the light period. Before the measurements, cells were concentrated to 4–10 × 10^6^ cells ml^−1^ at acclimation temperature by gentle vacuum filtration over polycarbonate filters (Isopore TSTP, 3 μm or RTTP, 1.2 μm; Isopore membranes, Merck, Darmstadt, Germany). In this process, the medium was successively exchanged with pH‐buffered DIC‐free culture medium (50 mM N,N‐bis(2‐hydroxyethyl)‐glycine, BICINE; pH_NBS_ of 7.85 or 8.15), and 8 ml were placed into an temperature‐controlled MIMS cuvette in the dark. Subsequently, 25 μmol kg^−1^ membrane‐impermeable dextrane‐bound sulfonamide (DBS; Synthelec, Lund, Sweden) was added, inhibiting any external carbonic anhydrase. Samples were continuously stirred to keep the cell suspension homogeneously mixed. To disentangle carbonate chemistry in the cuvette, inorganic carbon was added as ~1400 or ~2800 μmol kg^−1^ NaHCO_3_ to the DIC‐free medium, buffered at a pH of 8.15 or 7.85, yielding four different carbonate chemistry conditions (Fig. 1; Table 1): ‘Low DIC/High pH’ (L_DIC_H_pH_), ‘High DIC/High pH’ (H_DIC_H_pH_), ‘Low DIC/Low pH ‘(L_DIC_L_pH_) and ‘High DIC/Low pH’ (H_DIC_L_pH_). For each carbonate chemistry condition, photosynthetic and respiratory O_2_ and C_i_ fluxes were measured in consecutive light‐dark intervals (6 min per step), at two different light levels (180 and 700 μmol photons m^−2^ s^−1^).

**Table 1:** Carbonate chemistry during mass measurements of O_2_ and inorganic carbon (C_i_) fluxes in *Emiliania huxleyi*

Acclimation	Carbonate chemistry	L_DIC_H_pH_	H_DIC_H_pH_	L_DIC_L_pH_	H_DIC_L_pH_
2N LL	[CO_2_]	12.4 ± 0.6	22.0 ± 0.8	24.0 ± 1.5	43.7 ± 1.8
	[HCO_3_^−^]	1370 ± 70	2500 ± 90	1420 ± 80	2590 ± 110
	[H^+^]	9.5 ± 0.2	9.5 ± 0.2	18.4 ± 0.3	18.4 ± 0.3
2N HL	[CO_2_]	13.6 ± 0.9	22.7 ± 1.0	nd	47.4 ± 2.0
	[HCO_3_^−^]	1560 ± 110	2730 ± 130	nd	2730 ± 110
	[H^+^]	9.1 ± 0.3	8.9 ± 0.3	nd	18.8 ± 0.3
1N LL	[CO_2_]	10.6 ± 0.5	20.5 ± 0.3	17.1 ± 0.5	40.9 ± 2.8
	[HCO_3_^−^]	1200 ± 50	2400 ± 30	1050 ± 30	2460 ± 160
	[H^+^]	9.2 ± 0.1	9.2 ± 0.1	17.4 ± 0.5	18.1 ± 0.0
1N HL	[CO_2_]	10.5 ± 0.6	18.6 ± 1.4	17.9 ± 1.8	32.5 ± 2.5
	[HCO_3_^−^]	1150 ± 70	2160 ± 130	1090 ± 120	1940 ± 200
	[H^+^]	9.3 ± 0.1	9.2 ± 0.1	17.5 ± 0.4	18.1 ± 0.5

Concentrations of CO_2_ (μmol kg^−1^), HCO_3_^−^ (μmol kg^−1^) and H^+^ (nmol kg^−1^) were assessed by means of mass spectrometry (*n *=* *3; ± SD).

2N LL/HL, diploid life‐cycle stage acclimated to low/high light; 1N LL/HL, haploid life‐cycle stage acclimated to low/high light; L_DIC_H_pH_, low dissolved inorganic carbon (DIC)/high pH; H_DIC_H_pH_, high DIC/high pH; L_DIC_L_pH_, low DIC/low pH; H_DIC_L_pH_, high DIC/low pH; nd, not determined.

### Calculations of oxygen and carbon fluxes

#### Oxygen fluxes

Net photosynthesis (Phot*,* μmol kg^−1^ min^−1^) and respiration (Resp, μmol kg^−1^ min^−1^) were deduced from steady‐state O_2_ fluxes in the light and dark, respectively (Badger *et al*., 1994):

(Eqn 1) $$\text{Phot}={\frac{{\text{dO}}_{2}}{dt}}_{\mathrm{light}}$$

(Eqn 2) $$\text{Resp}=-{\frac{{\text{dO}}_{2}}{dt}}_{\mathrm{dark}}$$

#### Carbonate chemistry before light (BL)

For the calculation of the C_i_ fluxes, carbonate chemistry before and after the light phase was determined. [CO_2_]_BL_ (μmol kg^−1^) could be directly taken from measured signals, whereas [HCO_3_^−^]_BL_ (μmol kg^−1^) was calculated according to Badger *et al*. (1994):

(Eqn 3) $${\left[{\text{HCO}}_{3}^{-}\right]}_{\mathrm{BL}}=\frac{{\frac{{\text{dCO}}_{2}}{dt}}_{\mathrm{BL}}+k_{+}\left[{\text{CO}}_{2}\right]{}_{\mathrm{BL}}\;-\;\text{Resp}/\text{RQ}}{k_{-}}$$

(dCO_2_/d*t*_BL,_ steady‐state CO_2_ evolution in the dark (μmol kg^−1^ min^−1^); *k*_*+*_ and *k*_*—*_ , effective rate constants for the conversion of CO_2_ to HCO_3_^−^ (min^−1^) and vice versa; RQ, respiratory quotient of 1 (Burkhardt *et al*., 2001; Rost *et al*., 2007)). Following Schulz *et al*. (2007), we applied the calculated effective rate constants derived from the measured pH, temperature and salinity in our assays:

(Eqn 4) $$k_{-}=\;k_{-1}\left[H^{+}\right]+\;k_{-4}$$

(Eqn 5) $$k_{+}=k_{+1}+k_{+4}\;[OH^{-}]$$

([H^+^] and [OH^−^], concentrations of hydrogen and hydroxide ions, respectively (mol kg^−1^); *k*_*−1*_ *, k*_*+1*_ *, k*_*−4*_ and *k*_*+4*_, rate constants (Zeebe & Wolf‐Gladrow, 2001)). To assess [H^+^], known [DIC] (μmol kg^−1^) was added to cell‐free medium. From the resulting increase in [CO_2_] (μmol kg^−1^), the ratio of [DIC] : [CO_2_] and thus [H^+^] could be derived (Zeebe & Wolf‐Gladrow, 2001):

(Eqn 6) $$\left[H^{+}\right]=\frac{-K_{1}^{*}\;\left[{\text{CO}}_{2}\right]-\sqrt{{\left(K_{1}^{*}\left[{\text{CO}}_{2}\right]\right)}^{2}-4\left({\left(\left[{\text{CO}}_{2}\right]\right)}^{2}\;\left[\text{DIC}\right]\;K_{1}^{*}K_{2}^{*}\right)}}{2\left([{\text{CO}}_{2}\right]-\left[\text{DIC}\right])}$$

($K_{1}^{}*$ and $K_{2}^{}*$, stoichiometric equilibrium constants (Roy *et al*., 1993)). [DIC]_BL_ was derived as the sum of the C_i_ species, where carbonate ions ([CO_3_^2−^]_BL_) can be assumed to be in equilibrium with [HCO_3_^−^]_BL_ (Schulz *et al*., 2006):

(Eqn 7) $${\left[\text{DIC}\right]}_{\mathrm{BL}}=\left[{\text{CO}}_{2}\right]{}_{\mathrm{BL}}+\left(1+r\right){\left[{\text{HCO}}_{3}^{-}\right]}_{\mathrm{BL}}$$

The constant *r* hereby represents the pH‐dependent ratio between [HCO_3_^−^] and [CO_3_^2−^] (Zeebe & Wolf‐Gladrow, 2001; Schulz *et al*., 2007), which is defined as:

(Eqn 8) $$r=\frac{\left[{\text{CO}}_{3}^{2-}\right]}{\left[{\text{HCO}}_{3}^{-}\right]}=\;\frac{K_{2}^{}*}{\left[H^{+}\right]}$$

#### Carbonate chemistry at the end of light (EL)

[CO_2_]_EL_ (μmol kg^−1^) was directly obtained from measurements, whereas [HCO_3_^−^]_EL_ (μmol kg^−1^) was derived following Schulz *et al*. (2007):

(Eqn 9) $$\left[{\text{HCO}}_{3}^{-}\right]{}_{\mathrm{EL}}=\left({\left[\text{DIC}\right]}_{\mathrm{BL}}-{\left[\text{DIC}\right]}_{\mathrm{consumed}}-\;\left[{\text{CO}}_{2}\right]{}_{\mathrm{EL}}\right)/\left(1+r\right)$$

[DIC]_consumed_ represents the concentration of DIC that was consumed in the course of the light interval and was defined as the sum of DIC used for photosynthesis ([O_2_]_evolved_/PQ, μmol kg^−1^) and for calcification ([DIC]_CaCO3_, μmol kg^−1^):

(Eqn 10) $${\left[\text{DIC}\right]}_{\mathrm{consumed}}=\frac{\left[O_{2}\right]{}_{\mathrm{evolved}}}{\text{PQ}}+{\left[\text{DIC}\right]}_{{\mathrm{CaCO}}_{3}}$$

[O_2_]_evolved_ hereby represents the concentration of O_2_ that evolved over the course of the light phase and PQ is the photosynthetic quotient of 1.1 (Burkhardt *et al*., 2001; Rost *et al*., 2007). [DIC]_CaCO3_ was constrained by the measured ratio of particulate inorganic to organic carbon (PIC : POC) of the calcifying diploid life‐cycle stage under similar light treatments (1.4 at low‐light and 0.8 at high‐light acclimations; Rokitta & Rost, 2012) and was assumed to scale linearly with [O_*2*_]_evolved_ (e.g. Paasche, 1999). Additionally, calcite production was normalized to the photoperiod (Schulz *et al*., 2007):

(Eqn 11) $${\left[\text{DIC}\right]}_{{\mathrm{CaCO}}_{3}}=\frac{[O_{2}]\;\text{evolved}}{\text{PQ}}\;\times \frac{16\;\text{Phot}\;-\;8\;\text{Resp}}{16\;\text{Phot}}\times \;\frac{\text{PIC}}{\text{POC}}$$

In the noncalcifying haploid stage, PIC : POC was set to zero. Sensitivity analyses in which the PIC : POC were allowed to vary within typical uncertainties, revealed negligible effects on the calculated carbonate chemistry and photosynthetic fluxes.

#### Carbon fluxes

Knowing the carbonate chemistry, total net CO_2_ uptake (CO_2_up_total_, μmol kg^−1^ min^−1^) was inferred directly from the steady‐state CO_2_ drawdown in the light following Badger *et al*. (1994):

(Eqn 12) $${\text{CO}}_{2}{\text{up}}_{\mathrm{total}}=\;-\frac{{\text{dCO}}_{2}}{dt_{\;\;\mathrm{EL}}}-k_{+}\;\left[{\text{CO}}_{2}\right]{}_{\mathrm{EL}}+k_{-}\;\left[{\text{HCO}}_{3}^{-}\right]{}_{\mathrm{EL}}$$

Total CO_2_ uptake can be divided into one part used for photosynthesis (CO_2_up_PS_, μmol kg^−1^ min^−1^) and another part used for calcification (CO_2_up_CaCO3_ *,* μmol kg^−1^ min^−1^). As HCO_3_^−^ is the major external C_i_ source for calcification, we assumed that only 20% of calcification is supplied by external CO_2_ (Sikes *et al*., 1980; Paasche, 2001; Rost *et al*., 2002). Overall calcification was constrained by photoperiod‐normalized PIC : POC ratios and was assumed to scale linearly with the photosynthetic oxygen evolution:

(Eqn 13) $${\text{CO}}_{2}{\text{up}}_{{\mathrm{CaCO}}_{3}}=0.2\;\times \;\frac{\text{Phot}}{\text{PQ}}\;\times \;\frac{\text{PIC}}{\text{POC}}\;\times \;\frac{16\;\text{Phot}-8\;\text{Resp}}{16\;\text{Phot}}$$

Please note that, similar to PIC : POC ratios, errors in the assumption of the CO_2_ usage for calcification can affect the estimated photosynthetic fluxes by relative constant and small offsets, but do not change the overall observed regulation patterns in response to carbonate chemistry. Accounting for the CO_2_ uptake for calcification, CO_2_up_PS_ could be calculated as:

(Eqn 14) $${\text{CO}}_{2}{\text{up}}_{\mathrm{PS}}={\text{CO}}_{2}{\text{up}}_{\mathrm{total}}-{\text{CO}}_{2}{\text{up}}_{{\mathrm{CaCO}}_{3}}$$

Photosynthetic HCO_3_^−^ uptake (HCO_3_^−^up, μmol kg^−1^ min^−1^) was estimated as the difference between photosynthetic net C_i_ fixation (calculated as Phot PQ^−1^) and net CO_2_ uptake for photosynthesis:

(Eqn 15) $${\text{HCO}}_{3}^{-}\text{up}=\;\frac{\text{Phot}}{\text{PQ}}-{\text{CO}}_{2}{\text{up}}_{\mathrm{PS}}$$

Knowing the photosynthetic net CO_2_ uptake, its fraction of the overall net photosynthetic C_i_ uptake ( $f_{{\mathrm{CO}}_{2}}$; cf. Kottmeier *et al*., 2014) was derived as:

(Eqn 16) $$f_{{\mathrm{CO}}_{2}}={\text{CO}}_{2}{\text{up}}_{\mathrm{PS}}/\left(\frac{\text{Phot}}{\text{PQ}}\right)$$

#### Rate normalization

All rates were normalized to the amount of chlorophyll *a* (Chl*a*) in the concentrated samples. Known amounts of cell suspension were filtered onto cellulose nitrate filters (0.45 μm; Sartorius, Gottingen, Germany) that were instantly frozen in liquid nitrogen. After extraction in 90% acetone, Chl*a* content was determined fluorimetrically (TD‐700 fluorometer; Turner Designs, Sunnyvale, CA, USA) following the protocol of Knap *et al*. (1996).

### Statistics

All experiments were carried out in biological triplicates. Fluxes estimated for the different carbonate chemistry conditions and at the same incoming PFD were tested pairwise for significant differences applying two‐sided *t*‐tests. Effects were called significant when *P*‐values were ≤ 0.05. In the figures, such significant differences were indicated by different lower‐case characters (e.g. a and b). Values denoted by two letters (e.g. ab) represent data that are not significantly different from a or b.

## Results

**Figure 2:**
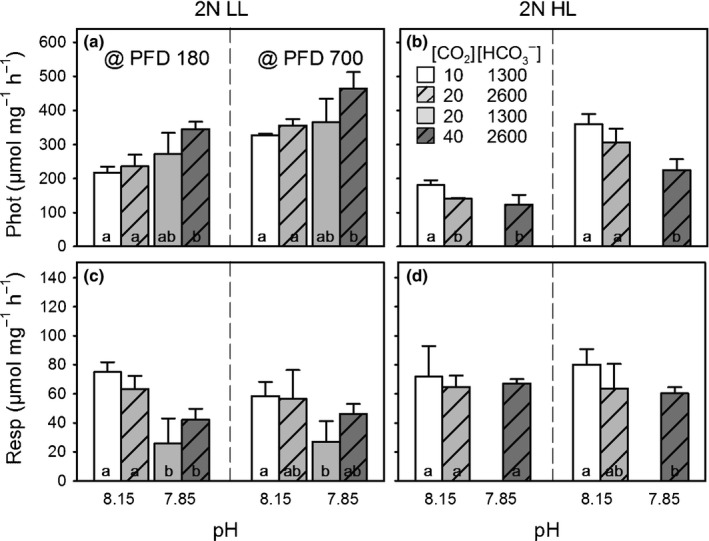
Short‐term modulations in photosynthetic and respiratory O_2_ fluxes of *Emiliania huxleyi* in response to low dissolved inorganic carbon (DIC)/high pH (L_DIC_H_pH_; white bars), as well as carbonation (H_DIC_H_pH_; dashed, light grey bars), acidification (L_DIC_L_pH_; light grey bars) and the combination of both (H_DIC_L_pH_; dashed, dark grey bars): Chl*a*‐normalized photosynthetic net O_2_ evolution (Phot; a, b) and respiration (Resp; c, d) were measured at low and high photon flux densities (PFD; 180 and 700* *μmol photons m^−2^ s^−1^). Data are shown for the diploid life‐cycle stage acclimated to low and high light (2N LL, 2N HL). Note: in 2N HL, no data for the L_DIC_L_pH_ condition were obtained. Error bar indicate mean ± SD (*n *=* *3). Different lower‐case characters indicate significant differences between the fluxes obtained at different carbonate chemistry conditions and same PFD.

**Figure 3:**
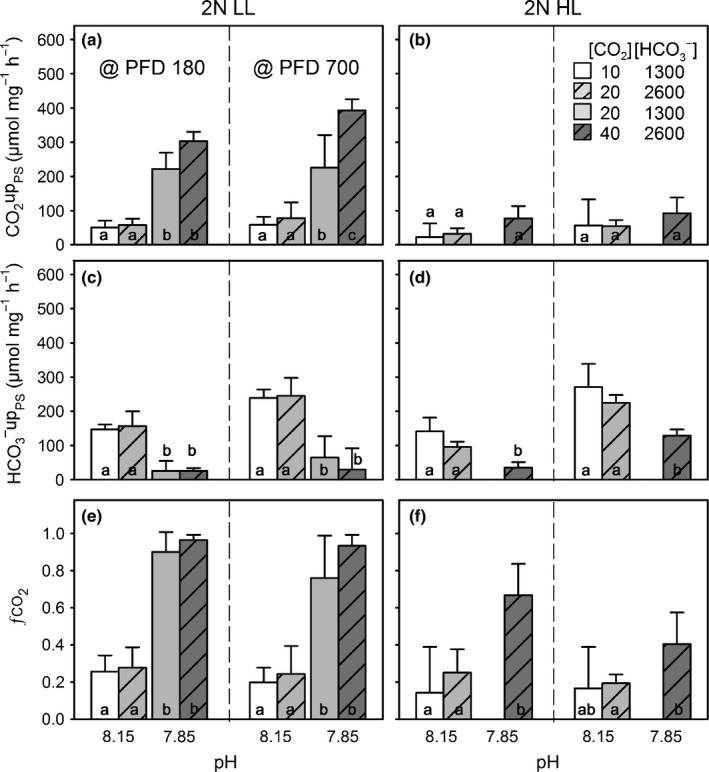
Short‐term modulations in external inorganic carbon (C_i_) fluxes of *Emiliania huxleyi* in response to low dissolved inorganic carbon (DIC)/high pH (L_DIC_H_pH_; white bars), as well as carbonation (H_DIC_H_pH_; dashed, light grey bars), acidification (L_DIC_L_pH_; light grey bars) and the combination of both (H_DIC_L_pH_; dashed, dark grey bars): Chl*a*‐normalized photosynthetic net CO_2_ uptake (CO_2_upt_PS_; a, b), photosynthetic HCO_3_^−^ uptake (HCO_3_^−^upt_PS_; c, d) and the fraction of overall photosynthetic net C_i_ uptake that is covered by net CO_2_ uptake ($f_{{\mathrm{CO}}_{2}}$; e, f) were measured at low and high photon flux densities (PFD; 180 and 700* *μmol photons m^−2^ s^−1^). Data are shown for the diploid life‐cycle stage acclimated to low and high light (2N LL, 2N HL). Note: in 2N HL, no data for the L_DIC_L_pH_ condition were obtained. Error bar indicate mean ± SD (*n *=* *3). Different lower‐case characters indicate significant differences between the fluxes obtained at different carbonate chemistry conditions and same PFD.

In the following, we describe treatment‐specific differences in short‐term responses to altered carbonate chemistry and light. For clarity, only the fluxes of the diplont are shown in Figs 2, 3. Fluxes of the haplont are given in Table 2.

**Table 2:** Short‐term modulations in photosynthetic O_2_ and inorganic carbon (C_i_) fluxes of *Emiliania huxleyi* in response to low dissolved inorganic carbon (DIC) and high pH (L_DIC_H_pH_), as well as carbonation (H_DIC_H_pH_), acidification (L_DIC_L_pH_) and the combination (H_DIC_L_pH_)

Acclimation	PFD	Carbonate chemistry	Phot (μmol mg^−1^ h^−1^)	Resp(μmol mg^−1^ h^−1^)	CO_2_upt_PS_(μmol mg^−1^ h^−1^)	HCO_3_^−^upt_PS_(μmol mg^−1^ h^−1^)	$f_{{\mathrm{CO}}_{2}}$
2N LL	180	L_DIC_H_pH_	217 ± 17	75 ± 7	51 ± 20	147 ± 15	0.26 ± 0.09
		H_DIC_H_pH_	236 ± 34	63 ± 9	58 ± 19	156 ± 43	0.28 ± 0.11
		L_DIC_L_pH_	271 ± 63	26 ± 17	221 ± 49	25 ± 29	0.90 ± 0.11
		H_DIC_L_pH_	345 ± 22	42 ± 8	303 ± 28	11 ± 9	0.96 ± 0.03
	700	L_DIC_H_pH_	327 ± 5	59 ± 10	59 ± 1	239 ± 1	0.20 ± 0.08
		H_DIC_H_pH_	355 ± 19	57 ± 20	78 ± 1	245 ± 1	0.24 ± 0.15
		L_DIC_L_pH_	365 ± 69	27 ± 14	226 ± 1	64 ± 1	0.76 ± 0.23
		H_DIC_L_pH_	464 ± 48	46 ± 7	393 ± 1	29 ± 1	0.93 ± 0.06
2N HL	180	L_DIC_H_pH_	181 ± 14	72 ± 21	23 ± 39	141 ± 40	0.14 ± 0.25
		H_DIC_H_pH_	141 ± 2	65 ± 8	32 ± 17	96 ± 15	0.25 ± 0.13
		L_DIC_L_pH_	nd	nd	nd	nd	nd
		H_DIC_L_pH_	124 ± 28	67 ± 3	77 ± 36	35 ± 16	0.67 ± 0.17
	700	L_DIC_H_pH_	360 ± 30	80 ± 11	56 ± 77	271 ± 68	0.17 ± 0.22
		H_DIC_H_pH_	307 ± 40	64 ± 17	55 ± 18	224 ± 23	0.19 ± 0.05
		L_DIC_L_pH_	nd	nd	nd	nd	nd
		H_DIC_L_pH_	225 ± 32	60 ± 4	93 ± 46	129 ± 18	0.40 ± 0.17
1N LL	180	L_DIC_H_pH_	147 ± 22	38 ± 8	1 ± 8	131 ± 14	0.00 ± 0.06
		H_DIC_H_pH_	173 ± 69	29 ± 7	28 ± 26	129 ± 39	0.15 ± 0.10
		L_DIC_L_pH_	202 ± 29	56 ± 12	58 ± 16	126 ± 19	0.31 ± 0.07
		H_DIC_L_pH_	185 ± 79	29 ± 3	55 ± 83	113 ± 17	0.24 ± 0.33
	700	L_DIC_H_pH_	223 ± 32	35 ± 4	−6 ± 8	207 ± 23	−0.03 ± 0.04
		H_DIC_H_pH_	240 ± 56	29 ± 5	7 ± 21	210 ± 41	0.02 ± 0.09
		L_DIC_L_pH_	286 ± 23	48 ± 11	26 ± 17	225 ± 25	0.11 ± 0.07
		H_DIC_L_pH_	273 ± 23	32 ± 4	38 ± 52	210 ± 37	0.14 ± 0.19
1N HL	180	L_DIC_H_pH_	119 ± 13	69 ± 4	−33 ± 7	141 ± 11	−0.31 ± 0.09
		H_DIC_H_pH_	113 ± 36	54 ± 8	−12 ± 26	114 ± 6	−0.18 ± 0.30
		L_DIC_L_pH_	136 ± 19	67 ± 19	0 ± 17	124 ± 24	0.00 ± 0.14
		H_DIC_L_pH_	148 ± 34	79 ± 20	−20 ± 17	154 ± 44	−0.14 ± 0.10
	700	L_DIC_H_pH_	246 ± 13	68 ± 8	−38 ± 10	261 ± 6	−0.17 ± 0.05
		H_DIC_H_pH_	196 ± 33	55 ± 9	−29 ± 24	207 ± 24	−0.18 ± 0.15
		L_DIC_L_pH_	236 ± 25	56 ± 8	−5 ± 8	208 ± 33	−0.02 ± 0.03
		H_DIC_L_pH_	274 ± 68	75 ± 9	−23 ± 2	272 ± 60	−0.10 ± 0.03

Chl*a*‐ normalized photosynthetic net O_2_ evolution (Phot) and respiration (Resp), photosynthetic net CO_2_ uptake (CO_2_upt_PS_), photosynthetic HCO_3_^−^ uptake (HCO_3_^−^upt_PS_) and the fraction of overall photosynthetic net C_i_ uptake that is covered by net CO_2_ uptake ($f_{{\mathrm{CO}}_{2}}$) were measured at low and high photon flux densities (PFD; 180 vs 700* *μmol photons m^−2^ s^−1^; *n *=* *3; ± SD).

2N LL/HL, diploid life‐cycle stage acclimated to low light/high light; 1N LL/HL, haploid life‐cycle stage acclimated to low/high light; L_DIC_H_pH_, low DIC/high pH; H_DIC_H_pH_, high DIC/high pH; L_DIC_L_pH_, low DIC/low pH; H_DIC_L_pH_, high DIC/low pH; nd, not determined.

### Oxygen fluxes

In both life‐cycle stages and light acclimations, net photosynthesis increased under increasing incoming light (Fig. 2a,b; PFD 180 vs 700), whereas dark respiration was generally independent of the light levels applied before the dark phase (Fig. 2c,d). The dependency on carbonate chemistry was stage and acclimation‐light specific (Fig. 2a–d; Table 2).

In the diplont acclimated to low light (2N LL), net photosynthesis was significantly stimulated under combined carbonation and acidification (H_DIC_L_pH_; Fig. 2a). This increase could not be attributed exclusively to carbonation or acidification, but appeared to be a product of both. Respiration in 2N LL decreased under H_DIC_L_pH_ (significantly only at PFD 180). This effect seemed to be driven by acidification, because the rates decreased significantly under both low‐pH conditions, but not with carbonation (Fig. 2c).

In the diplont acclimated to high light (2N HL), net photosynthesis was significantly impaired under H_DIC_L_pH_ (Fig. 2b). Also this effect seemed to be caused by carbonation and acidification together, although the drivers could not be identified statistically due to the lack of the L_DIC_L_pH_ data (Fig. 1; Tables 1, 2). Respiration in 2N HL was largely unaffected by carbonate chemistry (Fig. 2d).

In contrast to the diplont, photosynthesis and respiration in the low‐ and high‐light acclimated haplont (1N LL, 1N HL) were insensitive to the applied carbonate chemistry (Table 2).

### Carbon fluxes

In both life‐cycle stages and light acclimations, the higher C_i_ demands imposed by the higher incoming light levels during measurements were in most cases covered by additional HCO_3_^−^ uptake, whereas photosynthetic net CO_2_ uptake was largely unaffected by the incoming light (Fig. 3a–d; Table 2; PFD 180 vs PFD 700). The dependency of C_i_ fluxes on carbonate chemistry was clearly stage and acclimation‐light specific (Fig. 3; Table 2).

In 2N LL, the photosynthetic net CO_2_ uptake increased significantly under H_DIC_L_pH_ at both applied light levels, and these higher fluxes seemed to be driven mainly by acidification because CO_2_ uptake was strongly increased under both low‐pH conditions (Fig. 3a). Carbonation at high pH did not stimulate the CO_2_ uptake, whereas carbonation at low pH (LDICLpH vs HDICLpH) additionally increased CO_2_ uptake. HCO_3_^−^ uptake in 2N LL decreased significantly under H_DIC_L_pH_ (Fig. 3c). This decrease was clearly driven by acidification because HCO_3_^−^ uptake was decreased under both low‐pH conditions, independent of carbonation. The described opposing short‐term regulation of CO_2_ and HCO_3_^−^ uptake under H_DIC_L_pH_ caused significant shifts in $f_{{\mathrm{CO}}_{2}}$ from ~0.3 to ~0.9 (Fig. 3e).

In 2N HL, photosynthetic net CO_2_ uptake was relatively unaffected by carbonate chemistry (Fig. 3b). Similar to the low‐light acclimated cells, HCO_3_^−^ uptake for photosynthesis in 2N HL decreased significantly under H_DIC_L_pH_, presumably also driven by acidification (Fig. 3d). As a consequence of the relatively constant net CO_2_ uptake and the decreased HCO_3_^−^ uptake, $f_{{\mathrm{CO}}_{2}}$ increased significantly from ~0.2 to ~0.7 at a PFD of 180, whereas the increase was insignificant at 700 μmol photons m^−2^ s^−1^ (Fig. 3f).

In 1N LL, photosynthetic net CO_2_ uptake was close to zero and the photosynthetic HCO_3_^−^ uptake clearly dominated the C_i_ fluxes (Table 2). Both CO_2_ and HCO_3_^−^ fluxes were unaffected by carbonate chemistry, resulting in constant and low $f_{{\mathrm{CO}}_{2}}$ values (~0.1 on average; Table 2). In 1N HL, photosynthetic net CO_2_ uptake was negative, reflecting a net CO_2_ efflux alongside a high HCO_3_^−^ uptake (Table 2). Also here, CO_2_ and HCO_3_^−^ fluxes were unaffected by carbonate chemistry, resulting in constant and negative $f_{{\mathrm{CO}}_{2}}$ values (~−0.1).

## Discussion

In this study, we investigated *Emiliana huxleyi's* photosynthetic O_2_ and C_i_ fluxes and their short‐term modulations in response to changing carbonate chemistry and light. In the diploid life‐cycle stage (diplont), cellular fluxes were shown to be highly sensitive and to rapidly respond to the applied conditions. In the haploid stage (haplont), cellular fluxes were rather constant, even across large changes in carbonate chemistry.

### H^+^‐driven increase in CO_2_ uptake stimulates photosynthesis in low‐light acclimated diplonts

In the low‐light acclimated diplont (2N LL), rates of photosynthetic O_2_ evolution were in a similar range as measured earlier under comparable conditions (Nielsen, 1995; Rokitta & Rost, 2012). They stayed relatively constant under carbonation or acidification alone, but were strongly stimulated by combined carbonation and acidification (Fig. 2a). Ocean acidification (OA) has earlier been shown to affect cellular fluxes rapidly (Barcelos e Ramos *et al*., 2010; Kottmeier *et al*., 2014). An immediate stimulation in photosynthesis, if maintained over longer timescales, could therefore also explain the increase in particulate organic carbon (POC) production that is typically observed in OA‐acclimated coccolithophores (Raven & Crawfurd, 2012). Even though the applied carbonate chemistry matrix generally allowed for the distinction between the effects of carbonation and acidification, their differential effects were not evident from the observed O_2_ fluxes (Fig. 2a). Only by measuring the underlying C_i_ acquisition was it possible to identify the drivers behind the photosynthetic responses (Fig. 3a,c): Net CO_2_ uptake was strongly promoted under acidification as well as under combined carbonation and acidification (Fig. 3a), whereas HCO_3_^−^ uptake was strongly downscaled under these conditions (Fig. 3c). As the stimulation in net CO_2_ uptake under combined carbonation and acidification exceeded the impairing effect on HCO_3_^−^ uptake, the overall photosynthetic C_i_ uptake and consequently photosynthetic O_2_ evolution were increased under these conditions (Fig. 2a).

The transition from the active HCO_3_^−^ to diffusive CO_2_ uptake under short‐term acidification is in line with Kottmeier *et al*. (2014), who observed that *E. huxleyi* increases the relative fraction of CO_2_ usage when being exposed to high [CO_2_]/low pH over short timescales. Here we show that this shift is caused by a combination of increased CO_2_ uptake and decreased HCO_3_^−^ uptake. The increased CO_2_ usage is likely to decrease the energy demand of the cell, because transport of HCO_3_^−^ is considered more costly due to the molecule's negative charge and the large hydration envelope, properties that require an active transport (Burkhardt *et al*., 2001; Beardall & Raven, 2004; Holtz *et al*., 2015b). Indeed, we found stimulated photosynthesis and decreased respiration rates under acidification despite the same incoming light (Fig. 2a,c), indicating not only a more efficient CO_2_ supply at RubisCO, but also altered energy allocations under these conditions. Such energy reallocations under OA have earlier been attributed to shifts from reductive towards oxidative pathways (Rokitta *et al*., 2012).

The H^+^‐driven stimulation in CO_2_ uptake contradicts the ‘fertilizing effect’ of CO_2_ that is typically ascribed to OA. Contrary to the common notion that CO_2_ uptake for photosynthesis benefits from carbonation, it was here promoted mainly by acidification, at least over the short timescales applied. The higher CO_2_ uptake under combined carbonation and acidification compared to acidification alone indicated that high H^+^ levels generally increase the cellular CO_2_ uptake capacity. Yet, the higher CO_2_ availability was able to stimulate its uptake even further – carbonation and acidification acted synergistically. The H^+^‐driven decrease in cellular HCO_3_^−^ uptake, which occurred independent of the applied dissolved inorganic carbon (DIC) levels, indicated that the HCO_3_^−^ transport capacity is generally downscaled under acidification. Carbonation alone had no effect on HCO_3_^−^ uptake, suggesting that the transporters are substrate‐saturated at the applied [HCO_3_^−^] (~1300 and 2600 μmol kg^−1^). This is in line with a study by Rost *et al*. (2006) who measured the short‐term DIC‐dependency of photosynthesis at constant pH and showed that HCO_3_^−^ uptake in *E. huxleyi* was substrate‐saturated even below [HCO_3_^−^] of ~500 μmol kg^−1^.

The H^+^‐dependent regulations in C_i_ fluxes are likely to be similar after acclimation. Bach *et al*. (2011), for instance, acclimated *E. huxleyi* to carbonate chemistry conditions in which either CO_2_ or pH varied independently. They could show that POC production increases with DIC if pH is buffered to ~8.0, but it decreases with increasing DIC if pH decreases concomitantly. This suggests that the negative H^+^ effects on HCO_3_^−^ uptake are retained after acclimation. However, in contrast to our study, where no short‐term carbonation effects were measured, Bach and coworkers found stimulated POC and particulate inorganic carbon (PIC) production after acclimation to carbonation. Consequently, the cells were able to increase their C_i_ uptake when being exposed to these conditions over longer timescales, possibly by expressing more HCO_3_^−^ transporters. In order to examine how acclimation affects the sensitivity towards changing carbonate chemistry, future studies should investigate which short‐term effects manifest over longer timescales.

The strong H^+^ effects on CO_2_ and HCO_3_^−^ uptake rates, observed in the current study, must originate from processes at the cell membrane or inside the cell, such as electrochemical gradients, enzyme activities and C_i_ speciation (Mackinder *et al*., 2010; Suffrian *et al*., 2011; Taylor *et al*., 2011). The stimulated net CO_2_ uptake under acidification could, for example, be explained by pH‐dependent differences in membrane morphology (Leung *et al*., 2012), which may also affect the CO_2_ permeability. It could also be caused by pH‐dependent regulations of intracellular fluxes, for instance due to different enzyme activities, which may lead to a stronger inward CO_2_ gradient. The decreased HCO_3_^−^ uptake under acidification is apparently caused by a direct H^+^‐driven inhibition of HCO_3_^−^ transporters at the plasmalemma or chloroplast membrane. The diplont *E. huxleyi* expresses AE1 and AE2‐type Cl^−^/HCO_3_^−^ transporters of the Solute Carrier 4 (SLC4) family (Herfort *et al*., 2002; von Dassow *et al*., 2009; Mackinder *et al*., 2011; Rokitta *et al*., 2011; Bach *et al*., 2013). This enzyme family is well investigated in the context of renal acid/base regulation in mammals, where the activity of the anion exchangers has indeed been shown to be modulated by pH (Alper, 2006).

### H^+^‐driven decrease in HCO_3_^−^ uptake causes carbon‐limitation in high‐light acclimated diplonts

In high‐light acclimated diploid cells (2N HL), photosynthesis was inhibited under combined carbonation and acidification (Fig. 2b). This finding seems puzzling at first because in low‐light acclimated cells (2N LL), the same carbonate chemistry had a pronounced beneficial effect on photosynthesis (Fig. 2a). However, light‐dependent modulations in the sensitivity towards carbonate chemistry are well in line with other studies (e.g. Kranz *et al*., 2010; Gao *et al*., 2012; Rokitta & Rost, 2012; Jin *et al*., 2013; Hoppe *et al*., 2015). Rokitta & Rost (2012), for example, found that POC production in *E. huxleyi* is strongly stimulated when acclimated to OA and sub‐saturating light, but is relatively unaffected by OA under high light intensities.

Based on our flux measurements, we are able to provide an explanation for such differential OA sensitivities: In contrast to the low‐light acclimated cells, where CO_2_ uptake was strongly stimulated when being exposed to acidified conditions, CO_2_ uptake in the high‐light acclimated cells remained unaffected (Fig. 3b). Similar to the low‐light acclimated cells, HCO_3_^−^uptake in the high‐light acclimated cells was impaired under acidification (Fig. 3d). As a result, the overall C_i_ uptake and consequently photosynthetic O_2_ evolution were significantly decreased (Fig. 2b). The inability of high‐light acclimated cells to increase CO_2_ uptake may not only be the reason for the C_i_ shortage under short‐term acidification, but also explains why photosynthesis is often not stimulated after acclimation to OA (Raven & Crawfurd, 2012). However, a detrimental H^+^ effect on photosynthesis in *E. huxleyi* has not yet been observed after acclimation, indicating that either the decrease in HCO_3_^−^ uptake is less pronounced, or the increase CO_2_ uptake is more pronounced when cells are exposed to acidified conditions over an extended period of time.

The reduced capability of high‐light acclimated cells to increase CO_2_ uptake under acidification may derive from adjustments of their CO_2_‐concentrating mechanism (CCM) to the higher acclimation irradiance. *Emiliana huxleyi* was shown to increase HCO_3_^−^ uptake with increasing irradiance during flux measurements (Fig. 3d; 180 vs 700 PFD). This may indicate that high‐light acclimated cells also used a higher fraction of HCO_3_^−^ under the conditions, at which they were cultured (Rost *et al*., 2006). Cells operating CCMs that are based predominantly on HCO_3_^−^ uptake need to reduce the diffusive losses and therefore downregulate their CO_2_ permeability, for example by altering chloroplast morphology (Sukenik *et al*., 1987).

Recent studies on the combined effects of OA and light indicate that similar mechanisms, as here observed for *E. huxleyi*, also apply to other phytoplankton taxa. In diatoms, for example, growth was shown to increase significantly when cultured under OA and sub‐saturating light, whereas these responses were reversed under high light (Gao *et al*., 2012). Besides light intensity, light fluctuations also have been shown to significantly modulate OA effects (Jin *et al*., 2013; Hoppe *et al*., 2015). Hoppe and coworkers, for example, observed that photosynthesis stayed constant under OA and constant light, but decreased under OA and dynamic light. According to our data, OA may generally lower the HCO_3_^−^ uptake capacity of phytoplankton. Although this is apparently not detrimental under low and stable light conditions, the impaired HCO_3_^−^ uptake seems to have severe consequences under high and dynamic light conditions. Under the latter conditions, the phytoplankton cells are dependent primarily on HCO_3_^−^ transport, because the *high* Ci demand under high light and the *varying* Ci demand under dynamic light cannot be covered or adjusted fast enough by diffusive CO_2_ uptake. Owing to the impairment of HCO_3_^−^ transporters, these cells are thus more prone to C_i_ shortage at RubisCO under OA, even though external substrate concentrations are slightly elevated. When RubisCO becomes C_i_‐limited, the Calvin Cycle is a weaker electron sink, which can cause energetic overloads and higher costs associated with dissipation of energy and repair mechanisms (van de Poll *et al*., 2007; Gao *et al*., 2012; Jin *et al*., 2013; Hoppe *et al*., 2015). Thus, the high H^+^‐driven decrease in cellular HCO_3_^−^ uptake can explain why the energy transfer efficiency from photochemistry to biomass production is reduced under OA in combination with high or dynamic light conditions (Gao *et al*., 2012; Hoppe *et al*., 2015).

### Haplonts are insensitive to carbonate chemistry

The comparison of the two life‐cycle stages of *E. huxleyi* revealed that their modes of C_i_ acquisition strongly diverge. Photosynthetic and respiratory O_2_ fluxes in the haploid stage did not respond to the short‐term changes in carbonate chemistry (Table 2). Also CO_2_ and HCO_3_^−^ uptake were not affected by carbonation or acidification. This agrees with the results of acclimation studies that often found no or few changes in POC production and other cellular processes under OA (Rokitta & Rost, 2012; Kottmeier *et al*., 2014). The fact that HCO_3_^−^ uptake was unaffected by external H^+^ levels implies that the HCO_3_^−^ uptake mechanism of the haplont is different from the one of the diplont (Table 2). Indeed, there are transcriptomic datasets demonstrating that the two life‐cycle stages express different isoforms of HCO_3_^−^ transporters of the SLC4 family (von Dassow *et al*., 2009; Mackinder *et al*., 2011; Rokitta *et al*., 2011). Also, the haplont was shown to express stage‐specific subunits of a vacuolar H^+^ ATPase and other stage‐specific ion transporters, e.g. a Ca^2+^ ⁄ H^+^ antiporters, which may further explain the differential sensitivity towards H^+^ levels (von Dassow *et al*., 2009; Rokitta *et al*., 2011, 2012).

The consistently high HCO_3_^−^ usage of the haplont was not in line with the results of a ^14^C disequilibrium method, which estimated generally higher CO_2_ contributions and a strong dependency on [CO_2_]/pH (Kottmeier *et al*., 2014). This discrepancy may be attributed to the different key assumptions of the MIMS and/or the ^14^C disequilibrium methods. Regarding the MIMS method, we tested the consequences of potential offsets in key assumptions (e.g. variations in rate constants, PIC : POC, or photosynthetic quotient (PQ)) and found that typical uncertainties cannot explain the strong deviations between the methods. In contrast to the MIMS approach, the ^14^C disequilibrium technique does not yield actual CO_2_ and HCO_3_^−^ uptake rates, but estimates the relative CO_2_ uptake for photosynthesis (Lehman, 1971; Espie & Colman, 1986; Elzenga *et al*., 2000; Kottmeier *et al*., 2014). In this method, $f_{{\mathrm{CO}}_{2}}$ is assessed based on the curvature of the cellular photosynthetic ^14^C incorporation during a transient isotopic ^14^CO_2_ disequilibrium in the medium. In order to estimate $f_{{\mathrm{CO}}_{2}}$, the ^14^C‐incorporation is fitted with a model that is based on a number of parameters (Lehman, 1971; Espie & Colman, 1986). Some of these parameters, including kinetic constants, decay rates and the height of isotopic disequilibria remain error‐afflicted and are currently being re‐evaluated (S. Thoms *et al*. unpublished). Until these methodological discrepancies are better understood, the conflicting results for the haploid stage remain puzzling.

### Impaired HCO_3_^−^ uptake under acidification may affect calcification

**Figure 4:**
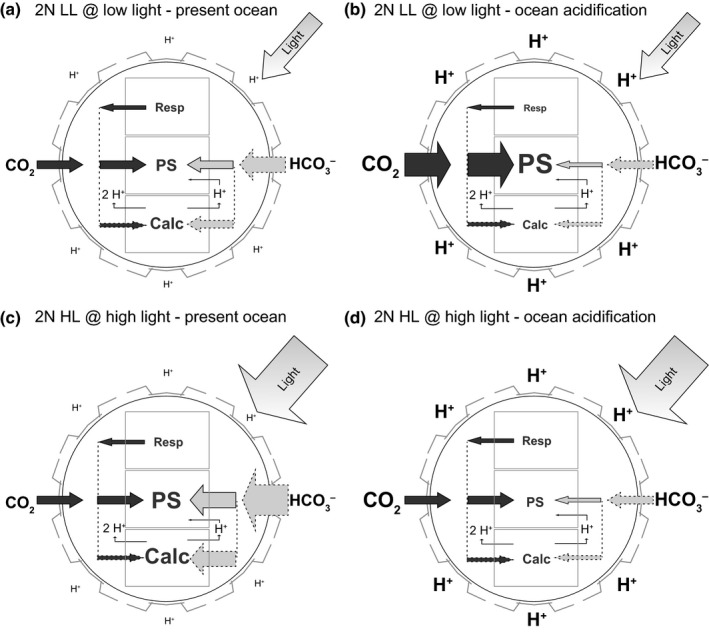
Schematic illustration of the ocean acidification (OA)‐dependent regulations in external inorganic carbon (C_i_) fluxes of diploid, low‐light acclimated (2N LL; a, b) and high‐light acclimated (2N HL; c, d) *Emiliania huxleyi* under acclimation light. Sizes of arrows with solid lines reflect the measured photosynthetic and respiratory fluxes of CO_2_ and HCO_3_^−^. Sizes of arrows with dashed lines reflect estimated fluxes. (a) Low‐light acclimated cells mainly use external HCO_3_^−^ as photosynthetic substrate under acclimation pH and achieve similar rates of calcification (Calc) and photosynthesis (PS). (b) When exposing low‐light acclimated cells to OA, cells increase CO_2_ uptake for photosynthesis, whereas HCO_3_^−^ uptake is downscaled due to the increased H^+^ levels. If HCO_3_^−^ fluxes into photosynthesis and calcification were downscaled to the same degree, photosynthesis would disproportionally increase over calcification. (c) High‐light acclimated cells perform higher rates of photosynthesis under acclimation light than low‐light acclimated cells. The increased C_i_ demand is covered by additional HCO_3_^−^ uptake. (d) When exposing high‐light acclimated cells to OA, they are not able to increase CO_2_ uptake rates, but nevertheless experience the H^+^‐driven decrease in HCO_3_^−^ uptake. As a consequence of the decreased overall C_i_ supply, photosynthesis and presumably calcification experience C_i_ shortage and thus decrease.

Although the strong negative H^+^ effects on photosynthetic HCO_3_^−^ uptake have not explicitly been described before, negative H^+^ effects on calcification are often discussed (Taylor *et al*., 2011; Fukuda *et al*., 2014; Bach *et al*., 2015; Cyronak *et al*., 2015). These inhibitory effects have often been attributed to changes in electrochemical gradients and the associated costs of H^+^ removal (Mackinder *et al*., 2010; Raven, 2011; Suffrian *et al*., 2011; Taylor *et al*., 2011). In agreement with Fukuda *et al*., 2014, we here found strong evidence that acidification impairs the HCO_3_^−^ uptake. Assuming that high H^+^ levels affect the transport of HCO_3_^−^ across the plasmalemma, the decreased uptake would not only influence photosynthesis, but also calcification. Based on flux measurements of this study, we illustrated the presumed cellular C_i_ fluxes in response to typical OA scenarios under different light acclimations (Fig. 4).

As HCO_3_^−^ fluxes into POC and PIC are similar in magnitude, calcification may serve intrinsic pH regulation (Sikes *et al*., 1980; Price *et al*., 2008; Raven, 2011). More specifically, when HCO_3_^−^ is used for photosynthesis, one H^+^ is consumed per fixed CO_2_, and when HCO_3_^−^ is used for calcification, one H^+^ is released per produced CaCO_3_ (Fig. 4; Holtz *et al*., 2015a). The additional photosynthetic CO_2_ uptake observed under acidification does not interfere with such a pH‐homeostatic behaviour. Thus, independent of the external carbonate chemistry and light conditions, the need to exchange H^+^ with the environment seems to be generally lower in the calcifying diplont of *E. huxleyi* (Fig. 4). This could provide the calcifying stage with an advantage over noncalcifying HCO_3_^−^ users (such as the haplont), which have to assure constant H^+^ uptake to compensate for alkalization during HCO_3_^−^‐based photosynthesis (Raven, 1986, 2011). This advantage may add to the diplont's success under bloom conditions, where seawater H^+^ levels can become low.

## Conclusions

In this study, we reveal a strong H^+^‐driven regulation of photosynthetic C_i_ fluxes of *E. huxleyi* that contradicts the commonly assumed ‘fertilizing effect’ of CO_2_. At typical present‐day conditions, HCO_3_^−^ was shown to be the major photosynthetic C_i_ source of both life‐cycle stages. High H^+^ levels were shown to rapidly inhibit the HCO_3_^−^ uptake and concomitantly to stimulate the CO_2_ uptake. This H^+^‐dependent inhibition in HCO_3_^−^ uptake serves as a mechanistic explanation for the typical OA‐dependent decline in calcification of coccolithophores and other marine calcifiers. Such an inhibition may be widespread among various phytoplankton taxa and also elucidates how the light‐use efficiency can decrease when phytoplankton communities are grown under OA in combination with high or fluctuating light intensities. Future research should investigate whether similar H^+^‐dependent flux regulations are also evident when cells are acclimated to altered conditions.

## Author contributions

D.M.K., S.D.R. and B.R. planned and designed the research. D.M.K. performed experiments and analysed the data. D.M.K., S.D.R. and B.R. interpreted the data and wrote the manuscript.

## Supporting information

Please note: Wiley Blackwell are not responsible for the content or functionality of any supporting information supplied by the authors. Any queries (other than missing material) should be directed to the *New Phytologist* Central Office.

**Table S1** Acclimation carbonate chemistry
