# Supplementary material for: Acidification, not carbonation, is the major regulator of carbon fluxes in the coccolithophore Emiliania huxleyi
Source: New Phytol. 2016 Feb 25;211(1):126–37. doi: 10.1111/nph.13885 (PMC5069628; doi:10.1111/nph.13885)
Supplement: Supplementary file 1 — Table S1 Acclimation carbonate chemistry [file NPH-211-126-s001.pdf]

New Phytologist

***New Phytologist* Supporting Information**

Article title: **Acidification, not carbonation, is the major regulator of carbon fluxes in the coccolithophore *Emiliania huxleyi***

Authors: Dorothee M. Kottmeier, Sebastian D. Rokitta and Björn Rost

Article acceptance date: 6 January 2016

The following Supporting Information is available for this article:

Table S1 Acclimation carbonate chemistry

Treatment	μ	pCO_2	pH_{NBS}	$[H^+]$	TA	DIC	$[CO_2]$	$[HCO_3^-]$	$[CO_3^{2-}]$
2N LL	0.69 ± 0.05	375 ± 33	8.20 ± 0.03	8.6 ± 0.8	2280 ± 19	2064 ± 17	14 ± 1	1896 ± 22	154 ± 10
2N HL	1.13 ± 0.05	376 ± 22	8.20 ± 0.02	8.5 ± 0.5	2288 ± 11	2072 ± 4	14 ± 1	1903 ± 8	155 ± 8
1N LL	0.68 ± 0.05	340 ± 3	8.25 ± 0.00	7.6 ± 0.1	2352 ± 3	2108 ± 1	13 ± 0	1921 ± 1	174 ± 1
1N HL	1.10 ± 0.07	343 ± 17	8.24 ± 0.02	7.7 ± 0.3	2349 ± 8	2107 ± 3	13 ± 1	1922 ± 9	173 ± 7
Reference		369 ± 12	8.21 ± 0.01	8.2 ± 0.2	2341 ± 4	2114 ± 5	14 ± 0	1937 ± 7	163 ± 4

Attained pCO_2 (µatm), pH, $[H^+]$ (nmol kg^{-1}), $[CO_2]$ (µmol kg^{-1}), $[HCO_3^-]$ (µmol kg^{-1}), $[CO_3^{2-}]$ (µmol kg^{-1}) were calculated based on measured DIC (µmol kg^{-1}) and TA (µmol kg^{-1}) using CO2sys (Pierrot *et al.*, 2006). Results are reported for 15°C ($n \geq 3$; ±SD). Input sizes for CO2sys calculations were salinity (31), pressure (0.1 dbar), as well as phosphate (7 µmol kg^{-1}) and silicate (7 µmol kg^{-1}) concentrations. Equilibrium constants by Mehrbach *et al.* (1973), refit by Dickson & Millero (1987) and dissociation constants for sulfuric acid by (Dickson, 1990) were applied.

References

Dickson AG. 1990. Standard potential of the reaction: AgCl(s) + ½ H_2(g) = Ag(s) + HCl(aq), and the standard acidity constant of the ion HSO_4^- in synthetic seawater from 273.15 to 318.15 K. *J. Chem. Thermodynamics* **22**: 113–127.

Dickson AG, Millero FJ. 1987. A comparison of the equilibrium constants for the dissociation of carbonic acid in seawater media. *Deep-Sea Res Pt I* **34**: 1733–1743.

Mehrbach C, Culberson CH, Hawley JE, Pytkowicz RM. 1973. Measurement of the apparent dissociation constants of carbonic acid in seawater at atmospheric pressure. *Limnology and Oceanography* **18**: 897–907.

Pierrot D, Lewis E, Wallace D. 2006. MS Excel program developed for CO_2 system calculations. *ORNL/CDIAC-105. Carbon Dioxide Information Analysis Center, Oak Ridge National Laboratory, US Department of Energy, Oak Ridge, TN, USA.*
